# Association Between Microscopic Hematuria and Proteinuria Remission in Adult Nephrotic Syndrome

**DOI:** 10.1016/j.xkme.2026.101333

**Published:** 2026-03-14

**Authors:** Ryuto Yoshida, Ryunosuke Mitsuno, Takashin Nakayama, Keita Hirano, Tomoaki Itoh, Dai Matsumoto, Ei Kusahana, Kenta Hoshi, Motoaki Komatsu, Yoichi Oshima, Seiei Iwabuchi, Kentaro Fujii, Yoshikazu Hara, Koji Futatsugi, Kyosei Nakamura, Takahisa Kawaguchi, Norifumi Yoshimoto, Aika Hagiwara, Akihito Hishikawa, Yasuyoshi Yamaji, Hirobumi Tokuyama, Marohito Murakami, Hiroto Matsuda, Chie Takimoto, Takashi Ando, Akinori Hashiguchi, Yuko Kaneko, Takashi Yokoo, Tatsuhiko Azegami, Kaori Hayashi

**Affiliations:** 1 Division of Nephrology, Endocrinology and Metabolism, Department of Internal Medicine, Keio University School of Medicine, Tokyo, Japan; 2 Division of Nephrology, Department of Internal Medicine, Japanese Red Cross Ashikaga Hospital, Tochigi, Japan; 3 Division of Nephrology and Hypertension, Department of Internal Medicine, Jikei University School of Medicine, Daisan Hospital, Tokyo, Japan; 4 Department of Nephrology, JCHO Saitama Medical Center, Saitama, Japan; 5 Division of Nephrology, Department of Internal Medicine, Tokyo Dental College, Ichikawa General Hospital, Chiba, Japan; 6 Division of Endocrinology, Metabolism and Nephrology, Department of Internal Medicine, Sano Kosei General Hospital, Tochigi, Japan; 7 Department of Nephrology, Tokyo Saiseikai Central Hospital, Tokyo, Japan; 8 Department of Nephrology, Keiyu Hospital, Kanagawa, Japan; 9 Department of Nephrology, Tachikawa Hospital, Tokyo, Japan; 10 Department of Nephrology, Kawasaki Municipal Ida Hospital, Kanagawa, Japan; 11 Department of Nephrology, Kawasaki Municipal Hospital, Kanagawa, Japan; 12 Department of Pathology, Keio University School of Medicine, Tokyo, Japan; 13 Division of Rheumatology, Department of Internal Medicine, Keio University School of Medicine, Tokyo, Japan; 14 Division of Nephrology and Hypertension, Department of Internal Medicine, Jikei University School of Medicine, Tokyo, Japan

**Keywords:** Focal segmental glomerulosclerosis, membranous nephropathy, microscopic hematuria, minimal change disease, nephrotic syndrome, podocytopathies, prognosis, proteinuria remission

## Abstract

**Rationale & Objective:**

Although the prognostic significance of hematuria has been established in various kidney diseases, few studies have investigated its role in nephrotic syndrome. This study aimed to characterize the prevalence and severity of microscopic hematuria at diagnosis and examine its association with proteinuria remission in adults with nephrotic syndrome.

**Study Design:**

Multicenter retrospective cohort study.

**Setting & Participants:**

Adults diagnosed with nephrotic syndrome who underwent native kidney biopsy between January 2012 and June 2022 at 10 institutions in Japan, with histological diagnoses of minimal change disease, focal segmental glomerulosclerosis, or membranous nephropathy.

**Predictors:**

Microscopic hematuria, assessed by urine sediment examination at the time of kidney biopsy and defined as positive when ≥5 red blood cells per high-power field were observed.

**Outcomes:**

Complete remission of proteinuria, defined as urine protein-creatinine ratio <0.3 g/g creatinine.

**Analytical Approach:**

The association between microscopic hematuria at diagnosis and complete remission was evaluated using the log-rank test and multivariable Cox proportional hazards models.

**Results:**

A total of 430 patients were enrolled. Microscopic hematuria was observed in 44.0% of patients overall, with prevalence varying by histological type: 30.0% in minimal change disease, 53.6% in focal segmental glomerulosclerosis, and 57.7% in membranous nephropathy. The rate of complete remission was significantly lower in the positive hematuria group (*P* = 0.001, log-rank test). Multivariable Cox regression analysis demonstrated that microscopic hematuria was independently associated with a lower likelihood of achieving complete remission (hazard ratio, 0.68; 95% confidence interval, 0.52-0.90). Furthermore, this negative impact on remission was dose-dependent, increasing with the severity of hematuria. The robustness of these findings was confirmed through distinct sensitivity analyses.

**Limitations:**

Changes in hematuria over time and recurrence were not assessed.

**Conclusions:**

In this cohort of adults with nephrotic syndrome, microscopic hematuria at diagnosis was independently associated with a lower rate of proteinuria remission.

## Plain-Language Summary

Although the prognostic significance of microscopic hematuria is established in many kidney diseases, its role in nephrotic syndrome has remained underrecognized. This prompted our investigation into its clinical utility. We conducted a multicenter retrospective study on 430 Japanese adults, analyzing their urine sediment at the time of their diagnostic kidney biopsy. Our primary finding was that microscopic hematuria was independently associated with a lower likelihood of achieving proteinuria remission. Moreover, this negative impact was dose-dependent, with more severe hematuria correlating with worse outcomes. This is clinically relevant as it positions microscopic hematuria, a readily available and noninvasive test, as a valuable biomarker for predicting treatment response in patients with nephrotic syndrome.

Hematuria is associated with the development^1^ and subsequent progression of chronic kidney disease.^2^, ^3^, ^4^ Although its prognostic significance remains a subject of debate, in glomerulonephritis, persistent microscopic hematuria is associated with kidney function decline in IgA nephropathy^5^ and with disease relapse in antineutrophil cytoplasmic antibody-associated glomerulonephritis.^6^ Interestingly, even in biopsy-confirmed diabetic nephropathy, where albuminuria is considered the main clinical feature, the presence of hematuria correlates with poor kidney prognosis.^7^

Conversely, in nephrotic syndrome, characterized by severe proteinuria and hypoalbuminemia, the role of hematuria has historically been less emphasized. A recent observational study using data from the Nephrotic Syndrome Study Network and Cure Glomerulopathy cohorts demonstrated that hematuria, detected by dipstick testing, was associated with lower remission rates and poor kidney outcomes, underscoring the importance of hematuria in podocytopathies, the primary causes of nephrotic syndrome.^8^ However, the prognosis of nephrotic syndrome varies by race and region, with reported differences between Asian and Western populations,^9^^,^^10^ highlighting the need for further validation in ethnically different cohorts. Furthermore, regarding the assessment of hematuria, the dipstick method used in a previous report^8^ may yield false-positive results, making it difficult to accurately assess the severity of hematuria.^11^ Therefore, in the present study, we assessed microscopic hematuria to more precisely quantify the presence and severity of red blood cells (RBCs) in urine and to better clarify their clinical significance.

We conducted a multicenter retrospective cohort study involving adult patients with nephrotic syndrome diagnosed with minimal change disease (MCD), focal segmental glomerulosclerosis (FSGS), or membranous nephropathy (MN). The aim of this study was to characterize the prevalence and severity of microscopic hematuria at the time of kidney biopsy across different histological types and to evaluate its association with proteinuria remission.

## Methods

### Study Overview and Participants

This multicenter retrospective cohort study was conducted across 10 institutions in Japan, comprising 2 university hospitals and 8 regional core hospitals. We enrolled adult patients (aged ≥18 years) diagnosed with nephrotic syndrome who underwent a native kidney biopsy between January 2012 and June 2022. Nephrotic syndrome was defined by the co-occurrence of severe proteinuria (either ≥3.5 g/d in a 24-hour urine collection or a spot urinary protein-creatinine ratio ≥3.5 g/g creatinine [cr]) and hypoalbuminemia (serum albumin ≤3.0 g/dL).^12^ The study focused on patients with histological diagnoses of MCD, FSGS, or MN. To permit a meaningful analysis of hematuria as a prognostic variable, we excluded glomerulonephritis (eg, IgA nephropathy), where microscopic hematuria is a near-universal feature. Exclusion criteria encompassed: (1) histological types other than MCD, FSGS, or MN; (2) insufficient biopsy material for a definitive diagnosis; and (3) unavailable data on microscopic hematuria at baseline. In addition, cases complicated by clinically apparent adaptive FSGS or diabetic kidney disease were excluded.

The study protocol received ethical approval from the Keio University School of Medicine Ethics Committee (approval number: 20241164) and was conducted in accordance with the principles of the Declaration of Helsinki. Informed consent was managed via an opt-out mechanism communicated through the institutional website.

### Measurements

Demographic data including age, sex, height, weight, body mass index (BMI), blood pressure, use of diuretics and comorbid conditions at the time of kidney biopsy were collected through electronic medical record review. The following laboratory information at the point closest to the kidney biopsy before treatment initiation was also obtained: serum albumin levels, serum and urinary levels of protein, creatinine, and urinary RBC counts. Estimated glomerular filtration rate (eGFR) was calculated using the following equation: 194 × cr^−1.094^× age^−0.287^ (× 0.739 if female).^13^ Urinary RBC counts were measured as part of routine clinical practice at the time of kidney biopsy, principally in accordance with the guidelines on urinary sediment examination procedures proposed by the Japanese Committee for Clinical Laboratory Standards.^14^ Within a few hours of collection, the sample was centrifuged at 500*g* for 5 minutes and manually assessed and classified into 3 grades of severity based on the number of cells per high-power field (HPF): negative (≤4 RBCs/HPF), mild (5-9 RBCs/HPF), severe (≥10 RBCs/HPF). Regarding the kidney biopsy, data on the histological classification, the total number of glomeruli, the number of globally sclerosed glomeruli, and the severity of interstitial fibrosis and tubular atrophy (IFTA) were collected. The proportion of global glomerulosclerosis was calculated, and IFTA was categorized into 4 groups: grade 0 (<10%), grade 1 (10%-25%), grade 2 (26%-50%), and grade 3 (>50%).^15^ The assessment of therapeutic efficacy in nephrotic syndrome was based on the amount of proteinuria; complete remission was defined as urine protein <0.3 g/g cr and partial remission as ≥0.3 g/g cr to <1.0 g/g cr.^12^ Study participants were followed up from kidney biopsy until complete remission, initiation of maintenance dialysis, or death, or until the end of the observation period (12 months after kidney biopsy). Additionally, any immunosuppressive agents used to treat nephrotic syndrome were documented.

### Outcomes

The primary outcome was the first achievement of complete remission, used to investigate the association between baseline microscopic hematuria (both presence and severity) and treatment response. In addition, we characterized the distribution and severity of microscopic hematuria according to histological type, age, and eGFR.

### Statistical Analyses

Study participants were stratified based on (1) the presence or absence of hematuria and (2) the severity of hematuria. For (1), participants were classified into a negative group (≤4 RBCs/HPF) and a positive group (≥5 RBCs/HPF).^1^^,^^2^^,^^7^ For (2), participants were categorized into 3 groups: a negative group (≤4 RBCs/HPF), a mild group (5-9 RBCs/HPF), and a severe group (≥10 RBCs/HPF). The clinical characteristics of each group were summarized accordingly.

Continuous variables were expressed as median with interquartile range (25th-75th percentiles), and categorical variables as numbers (percentages). The Mann–Whitney *U* test was used to compare continuous variables between 2 groups, and the Jonckheere–Terpstra test was applied for comparisons among 3 ordered groups. The χ^2^ test was employed to calculate the statistical significance of differences in categorical variables.

Survival curves were visualized using the Kaplan–Meier method and compared using the log-rank test. Cox regression analyses were used to investigate the association between the presence of microscopic hematuria and complete remission. Hazard ratios (HRs) with 95% confidence intervals (CIs) were determined in 3 models: model 1 (unadjusted), model 2 (adjusted for age, sex, and BMI), and model 3 (adjusted for hypertension [history of hypertension or systolic blood pressure ≥140 mm Hg at kidney biopsy], diabetes mellitus, eGFR, urinary protein levels, pathologic diagnosis, and the variables included in model 2). These covariates were selected based on clinical considerations regarding remission of nephrotic syndrome. We performed a subgroup analysis using model 3 stratified by each histological type, age, sex, BMI, urinary protein-creatinine ratio, eGFR, with or without hypertension and diabetes mellitus, and academic or regional hospital (to assess generalizability and explore potential selection bias). To assess whether the association between hematuria and complete remission differed across these subgroups, interaction terms were tested.

To confirm the robustness of the findings, we performed 6 sensitivity analyses. First, we excluded 64 patients who did not receive immunosuppressive agents. Second, we conducted a multivariable analysis selecting the proportion of global glomerulosclerosis instead of eGFR as a covariate. Third, we substituted IFTA for eGFR as an independent variable. Fourth, we redefined the outcome as the achievement of partial remission. Fifth, considering the potential competing risk of dialysis initiation and death, we employed the cumulative incidence function method and the subdistributional hazard model. Sixth, we used multivariate imputation by chained equations to deal with missing data (50 imputations); this imputation procedure considered all variables associated with the multivariable models.

A *P* value <0.05 was considered to be statistically significant. All statistical analyses were performed using Stata version 18 (StataCorp LLC).

## Results

### Patient Characteristics and the Prevalence and Severity of Microscopic Hematuria

**Figure 1:**
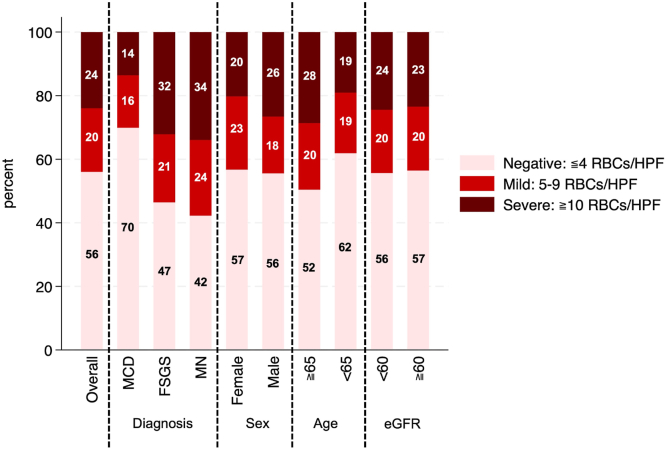
Distribution of microscopic hematuria severity across subgroups. A stacked 100% bar graph illustrating the severity of microscopic hematuria (negative/mild/severe) in patients with nephrotic syndrome, stratified by histological type (MCD, FSGS, MN), age group, and eGFR category. The graph highlights differences in hematuria severity among subgroups, with a higher prevalence of hematuria observed in patients with FSGS and MN compared to those with MCD. Abbreviations: eGFR, estimated glomerular filtration rate; FSGS, focal segmental glomerulosclerosis; HPF, high-power field; MCD, minimal change disease; MN, membranous nephropathy; RBC, red blood cell.

Of the 3,469 patients who underwent kidney biopsy, 812 were diagnosed with nephrotic syndrome. Among these, 382 patients were excluded: 342 due to histological types other than those specified (diabetic kidney disease [n = 98], IgA nephropathy [n = 53], lupus nephritis [n = 38], renal amyloidosis [n = 36], membranoproliferative glomerulonephritis [n = 29], and other diagnoses [n = 88]), 3 due to uninterpreted biopsy results, and 37 due to missing data. Consequently, 430 patients were included in the present study, with a median age of 66 years and 41.4% being female. The distribution of histological diagnoses was as follows: MCD in 206 patients (48%), FSGS in 56 patients (13%), and MN in 168 patients (39%) (Figure S1). The proportion of patients treated with corticosteroids was 93.2% (192 of 206) for MCD, 64.3% (36 of 56) for FSGS, and 80.4% (135 of 168) for MN. Furthermore, the proportion of patients who received other immunosuppressive agents was 26.7% (55 of 206) for MCD, 41.1% (23 of 56) for FSGS, and 48.8% (82 of 168) for MN. The severity of microscopic hematuria according to histological type, sex, age, and eGFR is shown in Figure 1. Overall, 44.0% of patients exhibited microscopic hematuria. Hematuria was observed in 30.0% of patients with MCD (62 of 206), 53.6% of those with FSGS (30 of 56), and 57.7% of those with MN (97 of 168). Patients in the negative group (≤4 RBCs/HPF) were younger and had a higher proportion of MCD, along with lower proportions of FSGS and MN, than those in the positive group (≥5 RBCs/HPF) (Table 1). Subsequently, the dataset was further stratified into 3 groups: the negative group (≤4 RBCs/HPF), the mild group (5-9 RBCs/HPF) and the severe group (≥10 RBCs/HPF), and the clinical characteristics of each group were summarized (Table S1).

**Table 1:** Characteristics of Study Patients Stratified by Presence of Microscopic Hematuria

Variable	Total (n = 430)	Positive Hematuria (n = 189)	Negative Hematuria (n = 241)	*P*
Age, y	66 (48-77)	69 (50-78)	64 (46–74)	0.008
Female	178 (41.4%)	77 (40.7%)	101 (41.9%)	0.81
Diagnosis				
MCD	206 (47.9%)	62 (32.8%)	144 (59.8%)	<0.001
FSGS	56 (13.0%)	30 (15.9%)	26 (10.8%)	<0.001
MN	168 (39.1%)	97 (51.3%)	71 (29.5%)	<0.001
Body mass index, kg/m^2^	23.7 (21.2-26.8)	23.6 (21.1-27.0)	23.8 (21.4-26.5)	0.78
Blood pressure, mm Hg				
Systolic	130 (118-147)	132 (121-148)	128 (114-146)	0.05
Diastolic	77 (69-87)	78 (70-87)	77 (68-86)	0.35
Use of diuretics	252 (58.6%)	110 (58.2%)	142 (58.9%)	0.88
Use of RASi	190 (44.2%)	84 (44.4%)	106 (44.0%)	0.92
Comorbid conditions				
Hypertension	261 (60.7%)	140 (58.1%)	121 (64.0%)	0.21
Diabetes	75 (17.4%)	33 (17.5%)	42 (17.4%)	0.99
Blood test				
Albumin, g/dL	1.9 (1.4-2.4)	1.9 (1.4-2.3)	1.8 (1.3-2.5)	0.90
Protein, g/dL	4.8 (4.3-5.6)	4.9 (4.4-5.5)	4.8 (4.2-5.7)	0.69
Creatinine, mg/dL	0.94 (0.74-1.34)	0.93 (0.76-1.42)	0.94 (0.73-1.23)	0.43
eGFR, mL/min/1.73 m^2^	58.9 (39.2-74.1)	58.8 (33.7-72.9)	59.3 (41.4-75.8)	0.27
Urine test				
Urinary protein, g/g cr	8.2 (5.3-11.7)	8.7 (5.6-12.5)	7.8 (5.1-11.1)	0.05
Kidney biopsy				
Global sclerosis ratio, %	10.0% (0.0%-23.8%)	9.7% (0.0%-25.0%)	10.5% (1.3%-23.7%)	0.76
IFTA, grade^a^	0 (0-1)	0 (0-1)	0 (0-1)	0.06
Grade 0	246 (57.8%)	151 (63.5%)	95 (50.8%)	
Grade 1	110 (25.9%)	46 (19.3%)	64 (34.2%)	
Grade 2	53 (12.5%)	30 (12.6%)	23 (12.3%)	
Grade 3	16 (3.8%)	11 (4.6%)	5 (2.7%)	
Immunosuppressants, n (%)				
Corticosteroid	363 (84.4%)	158 (83.6%)	205 (85.1%)	0.68
Others	160 (37.2%)	76 (40.2%)	84 (34.9%)	0.26

*Note:* Continuous variables are expressed as median with interquartile range (25th-75th percentiles) and categorical variables as numbers (percentages).

Abbreviations: cr, creatinine; eGFR, estimated glomerular filtration rate; FSGS, focal segmental glomerular sclerosis; IFTA, interstitial fibrosis and tubular atrophy; MCD, minimal change disease; MN, membranous nephropathy; RASi, renin angiotensin system inhibitor; RBC, red blood cell.

a Four grades: Grade 0 (<10%), Grade 1 (10%–25%), Grade 2 (26%–50%), and Grade 3 (>50%). Numbers do not sum to total due to missing IFTA data in 5 patients.

### Predictive Value of the Presence of Microscopic Hematuria for Proteinuria Remission

**Figure 2:**
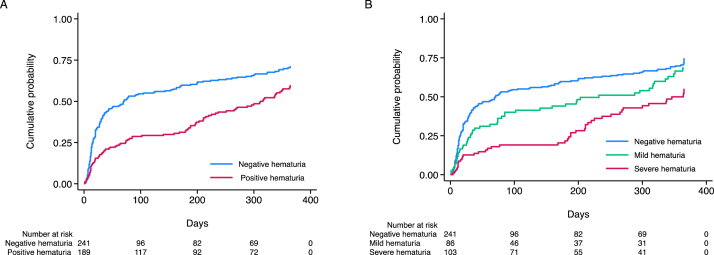
Kaplan–Meier curves for complete remission. (A) The curves illustrate the cumulative incidence of complete remission in patients with nephrotic syndrome, comparing the negative and positive hematuria groups at diagnosis. The log-rank test demonstrated a statistically significant difference between the 2 groups (*P* = 0.001). (B) The curves illustrate the cumulative incidence of complete remission in patients with nephrotic syndrome, comparing the negative, mild, and severe hematuria groups. A severity-dependent trend was observed, with the log-rank test indicating a statistically significant difference among the 3 groups (*P* < 0.001).

**Figure 3:**
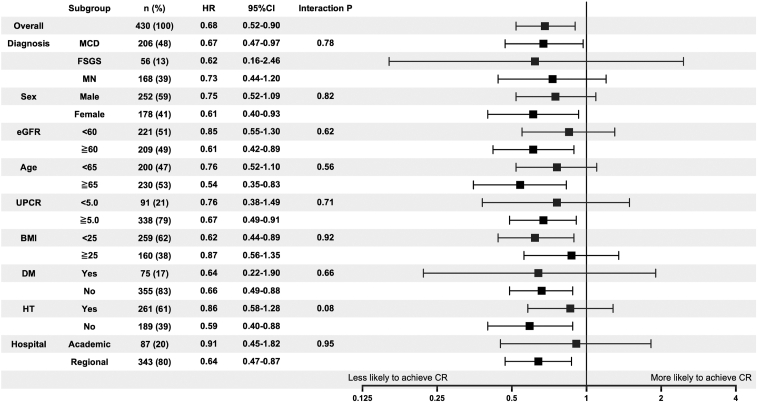
Subgroup analysis of Cox regression models stratified by patient characteristics. Forest plot showing HRs for complete remission across subgroups stratified by histological type (MCD, FSGS, MN), age, sex, BMI, UPCR, eGFR, presence of HT and DM, and hospital type (academic vs regional). The analysis highlights the association between microscopic hematuria and reduced remission rates across subgroups, emphasizing consistent trends regardless of patient characteristics. Microscopic hematuria was independently associated with lower remission rates in most subgroups, including patients with MCD. The subgroup analysis by BMI was performed on 419 patients with available data; 11 patients were excluded due to missing BMI values. Abbreviations: BMI, body mass index; CI, confidence interval; CR, complete remission; DM, diabetes mellitus; eGFR, estimated glomerular filtration rate; FSGS, focal segmental glomerulosclerosis; HR, hazard ratio; HT, hypertension; MCD, minimal change disease; MN, membranous nephropathy. UPCR, urinary protein-creatinine ratio.

During a median follow-up of 97 days (range, 20-350), 95 of 189 patients (50.3%) in the positive group and 161 of 241 patients (66.8%) in the negative group achieved complete remission. Among these 256 patients, 207 (81%) reached remission within 3 months, an additional 25 (10%) between 3 and 6 months, and the remaining 24 (9%) after 6 months. End-stage kidney disease or death occurred in 19 of 189 patients (10.1%) in the positive group and 10 of 241 patients (4.1%) in the negative group. Of these 29 patients who reached the endpoint, 9 initiated maintenance dialysis; their underlying diagnoses were FSGS (n = 5), MN (n = 3), and MCD (n = 1). Kaplan–Meier curves and the log-rank test indicated a significantly lower cumulative incidence of complete remission in the positive group (*P* = 0.001) (Figure 2A). In the overall population, unadjusted Cox regression analysis showed that microscopic hematuria was associated with a lower incidence of complete remission; this result remained consistent in Cox regression analysis adjusted for age, sex, and BMI. Multivariable Cox regression analysis (model 3) also demonstrated that the presence of microscopic hematuria was independently associated with lower complete remission rates (HR, 0.68; 95% CI, 0.52***-***0.90) (Table 2). The concordance index for the model 3 assessing microscopic hematuria was 0.78. The results of each subgroup analysis are shown in Figure 3. Even in the MCD group, multivariable Cox regression analysis (model 3) demonstrated that microscopic hematuria was independently associated with lower complete remission rates (HR, 0.67; 95% CI, 0.47-0.97).

**Table 2:** Results of Cox regression Analyses for Complete Remission by Presence of Microscopic Hematuria

Positive Hematuria	Complete Remission	
	HR (95% CI)	*P*
Overall		
Model 1	0.60 (0.47-0.77)	<0.001
Model 2	0.61 (0.47-0.79)	<0.001
Model 3	0.68 (0.52-0.90)	0.006

*Note:* Model 1: unadjusted; Model 2: age, sex, and body mass index; Model 3: Model 2 plus hypertension, diabetes, estimated glomerular filtration rate, urinary protein, and pathologic diagnosis.

Abbreviations: CI, confidence interval; HR, hazard ratio.

### Predictive Value of the Severity of Microscopic Hematuria for Proteinuria Remission

A total of 161 of 241 patients (67%) in the negative group, 52 of 86 patients (60%) in the mild group, and 43 of 103 patients (42%) in the severe group achieved complete remission. Kaplan–Meier curves and the log-rank test indicated that the complete remission rate differed according to the severity of hematuria (*P* < 0.001; Figure 2B). Multivariable Cox regression analysis (model 3) demonstrated a severity-dependent association. Compared with negative hematuria, severe hematuria was independently associated with a significantly lower rate of complete remission (HR, 0.55; 95% CI, 0.38-0.79). The association for mild hematuria was not statistically significant (HR, 0.84; 95% CI, 0.60-1.17) (Table 3).

**Table 3:** Results of Cox Regression Analyses for Complete Remission by Severity of Microscopic Hematuria

Models	Complete Remission		
	Negative	Mild	Severe
Model 1	1.0 (Reference)	0.78 (0.57-1.07) (*P* = 0.12)	0.47 (0.33-0.66) (*P* < 0.001)
Model 2	1.0 (Reference)	0.79 (0.57-1.09) (*P* = 0.15)	0.47 (0.33-0.67) (*P* < 0.001)
Model 3	1.0 (Reference)	0.84 (0.60-1.17) (*P* = 0.30)	0.55 (0.38-0.79) (*P* = 0.001)

*Note:* Data are shown as hazard ratio (95% confidence interval). Model 1: unadjusted; Model 2: age, sex, and body mass index; Model 3: Model 2 plus hypertension, diabetes, estimated glomerular filtration rate, urinary protein, and pathologic diagnosis.

### Sensitivity Analyses of the Predictive Value of Microscopic Hematuria for Proteinuria Remission

We conducted 6 sensitivity analyses. First, patients who did not receive immunosuppressive therapy were excluded; the primary findings remained consistent (HR, 0.71; 95% CI, 0.53-0.94) (Table S2). Second, in a multivariable Cox analysis replacing eGFR with the proportion of global glomerulosclerosis as a covariate, microscopic hematuria remained associated with a lower likelihood of achieving complete remission (HR, 0.68; 95% CI, 0.52-0.89) (Table S3). Third, multivariable analyses adjusted for IFTA consistently showed that hematuria was a predictor of a lower likelihood of achieving complete remission (HR, 0.72; 95% CI, 0.54-0.94) (Table S4). Fourth, when partial remission was defined as the outcome, the association with microscopic hematuria was attenuated and no longer statistically significant in the fully adjusted model (HR, 0.80; 95% CI, 0.62-1.02) (Table S5). Fifth, competing risk analysis demonstrated that hematuria was associated with a lower subdistribution hazard of subsequent complete remission (HR, 0.64; 95% CI, 0.48-0.87) (Figure S2; Table S6). Sixth, similar results were observed in a multivariable Cox regression model with multiple imputations for missing values (HR, 0.71; 95% CI, 0.54-0.93) (Table S7).

## Discussion

In this multicenter Japanese cohort of patients with nephrotic syndrome, we demonstrated that the presence of microscopic hematuria at diagnosis was independently associated with a lower likelihood of achieving proteinuria remission. Multiple sensitivity analyses confirmed the robustness of our primary findings. Despite its potential clinical relevance, the significance of hematuria in nephrotic syndrome has historically received limited research attention. This study highlights its importance and emphasizes the clinical utility of microscopic hematuria in nephrotic syndrome.

Hematuria is not an uncommon manifestation in nephrotic syndrome. However, its frequency and clinical significance in adult patients remain poorly characterized. A Turkish study^16^ reported hematuria in 35.8% (609 of 1,733) of patients with nephrotic syndrome. Regarding the frequency of hematuria by histological type, data from the Nephrotic Syndrome Study Network and Cure Glomerulopathy cohorts^8^ showed the lowest prevalence of hematuria detected by dipstick testing in patients with MCD (27%), followed by FSGS (35%) and MN (44%). Similarly, an Indian study^17^ of 410 adults reported hematuria in 21% of MCD, 35% of FSGS, and 25% of MN cases. Notably, a landmark study combining data from the Nephrotic Syndrome Study Network and Cure Glomerulopathy cohorts^8^ demonstrated that the presence of hematuria in nephrotic syndrome was significantly and independently associated with worse kidney-related outcomes, including both progressive loss of kidney function and a reduced likelihood of proteinuria remission. This prior research highlighted the clinical importance of hematuria in nephrotic syndrome, which had previously received little attention. However, the study had several limitations, including the use of dipstick testing for hematuria detection, potential selection bias due to timing discrepancies between study enrollment, diagnostic biopsy, and treatment initiation, as well as a limited representation of Asian participants, whose prognosis may differ from that of Western populations.^9^^,^^10^

The present study expands on the significance of hematuria in nephrotic syndrome by evaluating microscopic hematuria at the time of kidney biopsy in a Japanese cohort. Consistent with previous reports, microscopic hematuria was observed in 44.0% of patients overall, with a prevalence of 30.0% in MCD, 53.6% in FSGS, and 57.7% in MN. This study is novel in its detailed characterization of the prevalence of microscopic hematuria by histological type among adults with nephrotic syndrome. Moreover, consistent with previous reports, our cohort exhibited a higher proportion of patients achieving initial complete remission (59.5%, 256 of 430) compared to reports from Western populations.^8^ Despite this discrepancy, the presence of hematuria remained independently associated with lower remission rates in our Japanese nephrotic population, thereby providing external validation for prior findings. In addition to this validation, our study demonstrates novelty by showing a severity-dependent relationship between microscopic hematuria and reduced remission rates. In our multivariable model, the association between mild hematuria and complete remission did not reach statistical significance. However, the point estimate of the HR (0.84) indicated a trend toward a lower likelihood of remission, consistent with the findings for severe hematuria. This finding suggests a dose-dependent effect, where the prognostic weight of hematuria is predominantly carried by severe cases. Moreover, subgroup analyses revealed that microscopic hematuria was independently associated with lower complete remission rates, even among patients with MCD and those receiving immunosuppressive therapy.

The mechanisms underlying our primary findings may be explained as follows. The presence of hematuria in patients with nephrotic syndrome may reflect more severe filtration barrier damage, as glomerular hematuria indicates a defect in the glomerular basement membrane that allows RBCs to pass through the glomerular capillary into the urinary space. Additionally, as mentioned in previous reports, hematuria may be associated with genetic mutations that affect basement membrane structure. Specifically, data from the Toronto Glomerulonephritis registry identified pathologic variants of the *COL4A* gene that can present clinically as FSGS.^18^ Such underlying genetic defects could lead to a poorer response to standard immunosuppressive therapy, providing a strong potential mechanism for our finding that hematuria is associated with lower remission rates. Such genetic mutations affecting the integrity of the basement membrane may contribute to the lower remission rates observed in patients with microscopic hematuria. Another potential mechanism to consider is that persistent hematuria itself may have direct adverse effects on kidney prognosis. Indeed, previous studies have suggested that inflammation and oxidative stress induced by heme released from urinary RBCs may accelerate disease progression and worsen outcomes.^19^

Our study has clinical implications. Nephrotic syndrome represents a heterogeneous group of disorders classified based on histopathological findings observed in kidney biopsies^20^ that carries a high risk of life-threatening complications and progression to end-stage kidney disease.^21^, ^22^, ^23^ Given the variable clinical course of nephrotic syndrome, there remains a critical need for prognostic biomarkers linked to key kidney outcomes. Our findings align with previous reports suggesting that the presence of hematuria may serve as a prognostic marker in nephrotic syndrome. Furthermore, in the majority of cases in our study, microscopic hematuria was assessed before treatment initiation, and its presence remained a predictor of poor prognosis even among patients receiving immunosuppressive therapy. These results suggest that microscopic hematuria may also predict treatment response. In subgroup analysis, the presence of microscopic hematuria was associated with remission rates even within the same histological type (eg, among patients with MCD). This finding suggests that integrating the severity of microscopic hematuria with histological findings may enable a more detailed prediction of nephrotic syndrome prognosis. As a low-cost, noninvasive, and rapidly assessable parameter, microscopic hematuria holds potential as a valuable prognostic marker in nephrotic syndrome. Therefore, clinicians should recognize microscopic hematuria as an important prognostic factor not only in chronic kidney disease and glomerulonephritis but also potentially in nephrotic syndrome.

The strength of this study lies in its inclusion of both academic and regional core hospitals, enhancing the generalizability of our findings within Japan. In addition, the use of microscopic hematuria data obtained at the time of kidney biopsy, before therapeutic intervention, may better reflect the underlying disease pathophysiology. However, our study has several limitations. First, this was a retrospective cohort study. Despite adjusting numerous variables in the multivariable models, the possibility of residual confounding remains, including factors such as urolithiasis, urinary tract malignancies, timing of menstruation, and genetic mutations or variations. Hematuria may be attributable to factors other than nephrotic syndrome, particularly urological malignancies. Whether an adequate work-up for these possibilities was performed cannot be determined, which represents an important limitation. Second, the observation period is relatively short, preventing us from capturing the long-term impact on kidney function and achievement of complete remission after 12 months. Third, our study was based on a single measurement of hematuria at the time of kidney biopsy. We were unable to track longitudinal changes in hematuria, making it impossible to determine whether improvement in hematuria correlates with prognosis. Fourth, we evaluated only initial complete remission without assessing recurrence rates. Future research should focus on tracking changes in hematuria and monitoring long-term eGFR changes to address these limitations. Fifth, we did not account for ethnic or racial differences in disease phenotype. Sixth, the lack of a central pathology review may have led to inconsistencies in the evaluation of IFTA and diagnosis. Seventh, the absence of detailed data on the types and cumulative doses of immunosuppressive agents limited our ability to assess the potential impact of different treatment protocols on outcomes. Eighth, another limitation is the lack of genetic testing data. We were unable to systematically screen for underlying genetic conditions, such as Alport syndrome, which can present as FSGS with hematuria. Finally, we cannot exclude the possibility that some cases diagnosed as MCD with hematuria may have represented FSGS that was missed due to sampling error.

In conclusion, microscopic hematuria at diagnosis independently predicts lower rates of proteinuria remission in Japanese adults with nephrotic syndrome. Although often overlooked in clinical decision making, microscopic hematuria may serve as a valuable prognostic indicator in patients with nephrotic syndrome.

## Footnotes

Supplementary File (PDF)
**Figure S1:** Flowchart of participant selection. **Figure S2:** Competitive risk-adjusted cumulative incidence curves for complete remission. **Table S1:** Characteristics of Study Patients Stratified by Severity of Microscopic Hematuria. **Table S2:** Results of Cox Regression Analyses for Complete Remission (Only Patients Who Received Immunosuppressive Therapy). **Table S3:** Results of Cox Regression Analyses for Complete Remission (Adjusted for Proportion of Global Sclerosis Instead of Estimated Glomerular Filtration Rate). **Table S4:** Results of Cox Regression Analyses for Complete Remission (Adjusted for Proportion of Interstitial Fibrosis and Tubular Atrophy Instead of Estimated Glomerular Filtration Rate). **Table S5:** Results of Cox Regression Analyses for Partial Remission. **Table S6:** Results of Cox Regression Analyses for Complete Remission (Subdistributional Hazard Model). **Table S7:** Results of Cox Regression Analyses for Complete Remission (Multiple Imputations for Missing Values).
