# Supplementary material for: Association Between Microscopic Hematuria and Proteinuria Remission in Adult Nephrotic Syndrome
Source: Kidney Med. 2026 Mar 14;8(5):101333. doi: 10.1016/j.xkme.2026.101333 (PMC13092668; doi:10.1016/j.xkme.2026.101333)
Supplement: Supplementary File (PDF) — Figures S1, S2; Tables S1-S7. [file mmc1.pdf]

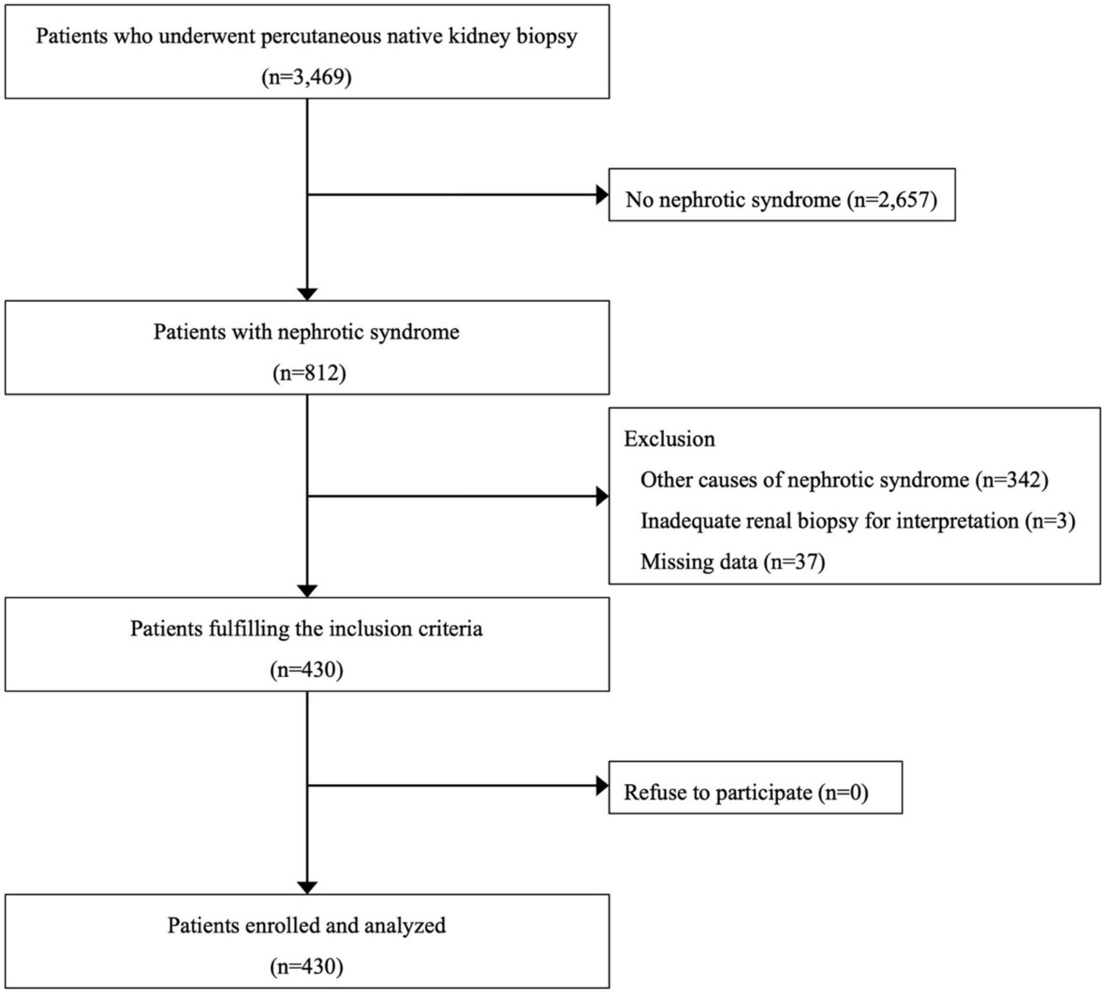

Figure S1. Flowchart of Participant Selection. Flowchart illustrating the inclusion and exclusion criteria for the study cohort.

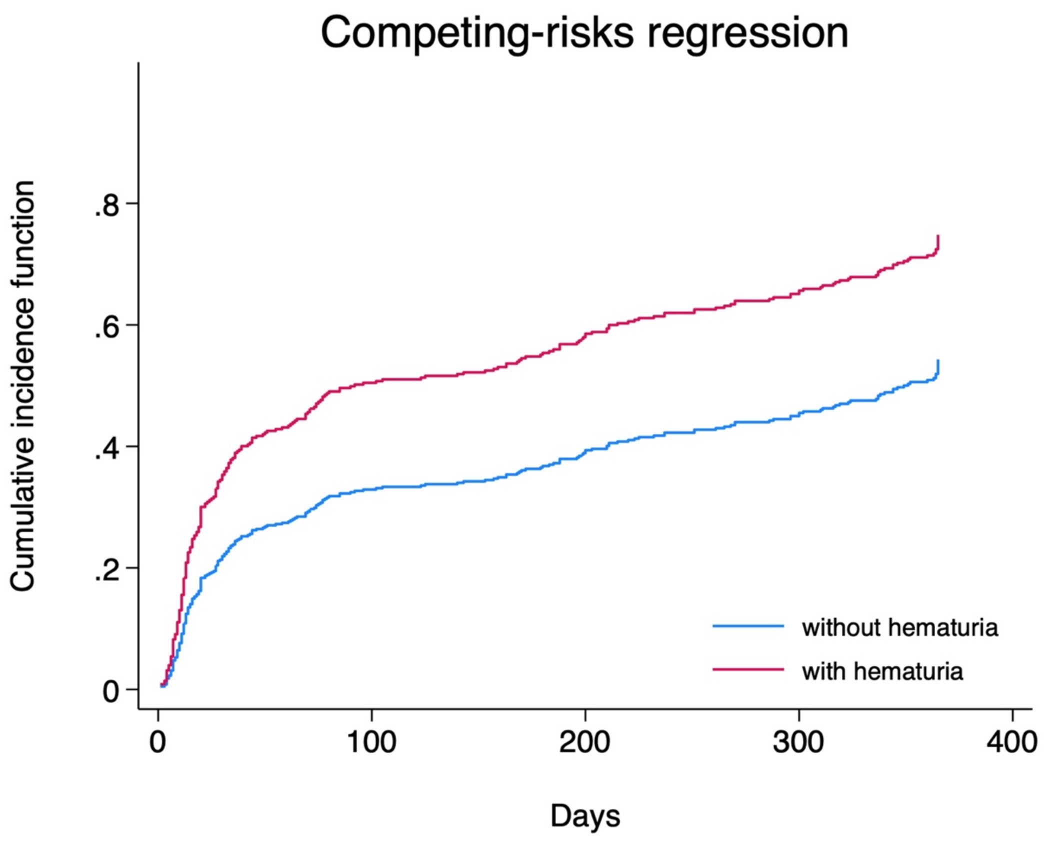

Figure S2. Competitive Risk-Adjusted Cumulative Incidence Curves for Complete Remission. Cumulative incidence curves illustrating the probability of achieving complete remission, accounting for competing risks such as initiation of maintenance dialysis or death. The analysis compares the negative hematuria group and the positive hematuria group. Results demonstrate a lower cumulative incidence of remission in the hematuria group, even after adjusting for competing risks.

Table S1. Characteristics of study patients stratified by severity of microscopic hematuria

Variable	Overall (n=430)			
	Negative (n=241)	Mild (n=86)	Severe (n=103)	P value
Age, year	64 (46–74)	69 (47–77)	70 (57–79)	0.003
Female, n (%)	101 (41.9)	41 (47.7)	36 (35.0)	0.20
Diagnosis, n (%)				
MCD	144 (59.8)	34 (39.5)	28 (27.2)	< 0.001
FSGS	26 (10.8)	12 (14.0)	18 (17.5)	< 0.001
MN	71 (29.5)	40 (46.5)	57 (55.3)	< 0.001
Body mass index, kg/m^2	23.8 (21.1–26.9)	23.0 (21.2–26.8)	23.8 (20.9–27.6)	0.64
Blood pressure, mmHg				
Systolic	128 (114–146)	131 (121–144)	136 (122–152)	0.02
Diastolic	77 (68–86)	78 (70–87)	78 (70–87)	0.27
Use of diuretics, n (%)	142 (58.9)	46 (53.5)	64 (62.1)	0.48
Use of RASi, n (%)	106 (44.0)	36 (41.9)	48 (46.6)	0.80
Comorbidities, n (%)				
Hypertension	140 (58.1)	49 (57.0)	72 (69.9)	0.09
Diabetes	42 (17.4)	10 (11.6)	23 (22.3)	0.16
Blood test				
Albumin, g/dl	1.8 (1.3–2.5)	1.8 (1.4–2.3)	1.9 (1.4–2.4)	0.69

Protein, g/dl	4.8 (4.2–5.7)	4.9 (4.4–5.4)	4.9 (4.4–5.6)	0.61
Creatinine, mg/dl	0.94 (0.73–1.23)	0.91 (0.76–1.27)	1.00 (0.73–1.66)	0.27
eGFR, ml/min/1.73 m^2	59.3 (41.4–75.8)	59.0 (39.4–75.3)	58.8 (29.3–71.8)	0.16
Urine test				
Urinary protein, g/gCr	7.8 (5.1–11.1)	8.2 (5.0–11.7)	9.1 (6.0–12.9)	0.02
Kidney biopsy				
Global sclerosis ratio, %	10.5 (1.3–23.7)	6.6 (0–21.9)	12.7 (3.8–27.8)	0.78
IFTA, grade*	0 (0–1)	0 (0–1)	0 (0–1)	0.03
Grade 0	151 (63.5)	47 (54.7)	48 (47.5)	
Grade 1	46 (19.3)	29 (33.7)	35 (34.7)	
Grade 2	30 (12.6)	9 (10.5)	14 (13.9)	
Grade 3	11 (4.6)	1 (1.2)	4 (4.0)	
Immunosuppressants, n (%)				
Corticosteroid	205 (85.1)	69 (80.2)	89 (86.4)	0.47
Others	84 (34.9)	28 (32.6)	48 (30.0)	0.07

Abbreviations: eGFR, estimated glomerular filtration rate; FSGS, focal segmental glomerular sclerosis; IFTA, interstitial fibrosis and tubular atrophy; MCD, minimal change disease; MN, membranous nephropathy; RASi, renin angiotensin system inhibitor; RBC, red blood cell. *Four grades: Grade 0 (<10%), Grade 1 (10%–25%), Grade 2 (26%–50%), and Grade 3 (>50%). *Numbers do not sum to total due to missing IFTA data in five patients. Continuous variables were expressed as median with interquartile range (25th–75th percentiles), and categorical variables as numbers (percentages).

Table S2. Results of Cox regression analyses for complete remission (only patients who received immunosuppressive therapy)

Positive hematuria	Complete remission	
	HR (95% CI)	P value
Overall		
Model 1	0.61 (0.47–0.80)	< 0.001
Model 2	0.60 (0.46–0.79)	< 0.001
Model 3	0.71 (0.53–0.94)	0.02

Model 1: unadjusted; Model 2: age, sex, and body mass index; Model 3: Model 2 plus hypertension, diabetes, estimated glomerular filtration rate, urinary protein, and pathological diagnosis.
Abbreviations: CI, confidence interval; HR, hazard ratio

Table S3. Results of Cox regression analyses for complete remission (adjusted for proportion of global sclerosis instead of estimated glomerular filtration rate)

Positive hematuria	Complete remission	
	HR (95% CI)	P value
Overall		
Model 1	0.60 (0.47–0.77)	< 0.001
Model 2	0.61 (0.47–0.79)	< 0.001
Model 3	0.68 (0.52–0.89)	0.005

Model 1: unadjusted; Model 2: age, sex, and body mass index; Model 3: Model 2 plus hypertension, diabetes, proportion of global sclerosis, urinary protein, and pathological diagnosis.
Abbreviations: CI, confidence interval; HR, hazard ratio

Table S4. Results of Cox regression analyses for complete remission (adjusted for proportion of interstitial fibrosis and tubular atrophy instead of estimated glomerular filtration rate).

Positive hematuria	Complete remission	
	HR (95% CI)	P value
Overall		
Model 1	0.60 (0.47–0.77)	< 0.001
Model 2	0.61 (0.47–0.79)	< 0.001
Model 3	0.72 (0.54–0.94)	0.02

Model 1: unadjusted; Model 2: age, sex, and body mass index; Model 3: Model 2 plus hypertension, diabetes, proportion of interstitial fibrosis and tubular atrophy, urinary protein, and pathological diagnosis.
Abbreviations: CI, confidence interval; HR, hazard ratio

Table S5. Results of Cox regression analyses for partial remission.

Positive hematuria	Partial remission	
	HR (95% CI)	P value
Overall		
Model 1	0.68 (0.54–0.86)	0.001
Model 2	0.71 (0.56–0.89)	0.004
Model 3	0.80 (0.62–1.02)	0.07

Model 1: unadjusted; Model 2: age, sex, and body mass index; Model 3: Model 2 plus hypertension, diabetes, estimated glomerular filtration rate, urinary protein, and pathological diagnosis.
Abbreviations: CI, confidence interval; HR, hazard ratio

Table S6. Results of Cox regression analyses for complete remission (subdistributional hazard model)

Positive hematuria	Complete remission	
	HR (95% CI)	P value
Overall		
Model 1	0.57 (0.44–0.73)	< 0.001
Model 2	0.58 (0.45–0.76)	< 0.001
Model 3	0.64 (0.48–0.87)	< 0.001

Model 1: unadjusted; Model 2: age, sex, and body mass index; Model 3: Model 2 plus hypertension, diabetes, estimated glomerular filtration rate, urinary protein, and pathological diagnosis.
Abbreviations: CI, confidence interval; HR, hazard ratio

Table S7. Results of Cox regression analyses for complete remission (multiple imputations for missing values)

Positive hematuria	Complete remission	
	HR (95% CI)	P value
Overall		
Model 1	0.60 (0.47–0.77)	< 0.001
Model 2	0.61 (0.47–0.79)	< 0.001
Model 3	0.71 (0.54–0.93)	0.01

Model 1: unadjusted; Model 2: age, sex, and body mass index; Model 3: Model 2 plus hypertension, diabetes, estimated glomerular filtration rate, urinary protein, and pathological diagnosis.
Abbreviations: CI, confidence interval; HR, hazard ratio
